# Effects of supplemental creatine and guanidinoacetic acid on spatial memory and the brain of weaned Yucatan miniature pigs

**DOI:** 10.1371/journal.pone.0226806

**Published:** 2020-01-06

**Authors:** Jason L. Robinson, Laura E. McBreairty, Rebecca A. Ryan, Raniru Randunu, Carolyn J. Walsh, Gerard M. Martin, Janet A. Brunton, Robert F. Bertolo

**Affiliations:** 1 Department of Biochemistry, Memorial University of Newfoundland, St. John’s, Newfoundland and Labrador, Canada; 2 Department of Psychology, Memorial University of Newfoundland, St. John’s, Newfoundland and Labrador, Canada; University of Illinois, UNITED STATES

## Abstract

The emergence of creatine as a potential cognitive enhancement supplement for humans prompted an investigation as to whether supplemental creatine could enhance spatial memory in young swine. We assessed memory performance and brain concentrations of creatine and its precursor guanidinoacetic acid (GAA) in 14-16-week-old male Yucatan miniature pigs supplemented for 2 weeks with either 200 mg/kg∙d creatine (+Cr; n = 7) or equimolar GAA (157 mg/kg∙d) (+GAA; n = 8) compared to controls (n = 14). Spatial memory tests had pigs explore distinct sets of objects for 5 min. Objects were spatially controlled, and we assessed exploration times of previously viewed objects relative to novel objects in familiar or novel locations. There was no effect of either supplementation on memory performance, but pigs successfully identified novel objects after 10 (p < 0.01) and 20 min (p < 0.01) retention intervals. Moreover, pigs recognized spatial transfers after 65 min (p < 0.05). Regression analyses identified associations between the ability to identify novel objects in memory tests and concentrations of creatine and GAA in cerebellum, and GAA in prefrontal cortex (p < 0.05). The concentration of creatine in brain regions was not influenced by creatine supplementation, but GAA supplementation increased GAA concentration in cerebellum (p < 0.05), and the prefrontal cortex of +GAA pigs had more creatine/g and less GAA/g compared to +Cr pigs (p < 0.05). Creatine kinase activity and maximal reaction velocity were also higher with GAA supplementation in prefrontal cortex (p < 0.05). In conclusion, there appears to be a relationship between memory performance and guanidino compounds in the cerebellum and prefrontal cortex, but the effects were unrelated to dietary supplementation. The cerebellum is identified as a target site for GAA accretion.

## Introduction

Creatine (*N-*aminoiminomethyl-*N*-methylglycine) is a guanidino compound that acts primarily as an energy buffer in vertebrates upon phosphorylation to phosphocreatine by the enzyme creatine kinase (EC 2.7.3.2). Dietary sources of creatine are primarily animal products, or creatine monohydrate supplementation. Creatine is among the most popular dietary supplements in the world and is consumed to increase intramuscular availability of phosphocreatine [1]. Interestingly, creatine supplementation has also been effective at enhancing cognitive performance in vegetarians [2,3] and in sleep-deprived younger [4] and older adults [5]. The benefit of supplemental creatine is thought to be due to phosphocreatine availability in the brain, which supports memory consolidation by providing ATP [6]. However, other studies in healthy adults have found no cognitive benefit to creatine supplementation [7] and possibly, the cognitive effects of creatine supplementation may only be apparent during stress or when exogenous supply or endogenous synthesis is restricted. Indeed, studies in rats show improved efficiency with spatial working memory during chronic stress following a single creatine dose [8]. Whereas mice chronically supplemented with creatine elicited better performance on a variety of memory tasks and had improved mitochondrial energetics [9]. Many have argued that creatine has neuroprotective effects that may be beneficial after mild brain trauma [10], or in neurodegenerative disease [11]. However, the distribution of creatine in the brain and the capacity of oral supplementation to affect creatine metabolism in brain is unknown.

Creatine synthesis commences in the pancreas and kidneys where arginine:glycine amidinotransferase (AGAT; EC 2.1.4.1) catalyzes the formation of guanidinoacetic acid (GAA) and ornithine from arginine and glycine [12,13]. GAA is then transported into the liver by γ-aminobutyric acid transporter 2 (GAA2) [14], where it is methylated to creatine by guanidinoacetate N-methyltransferase (GAMT; EC 2.1.1.2). Creatine is transported into extrahepatic tissues by creatine transporter-1 (CT1) that is coded by the gene SLC6A8. CT1 also mediates GAA and phosphocreatine transport [15,16], but whether CT1 contributes meaningfully to GAA and creatine distribution in the brain is unclear [17]. The ability to synthesize, distribute, and absorb creatine is critical for normal brain function as evidenced by a substantial body of literature implicating perturbed creatine metabolism in a number of neurological disorders [18,19].

Intracranial production of creatine and GAA is also likely, and there is evidence that GAA and creatine interact with the GABA receptor in brain of mice [20] and chicks [21]. Within the central nervous system, AGAT and GAMT can be found in almost every cell type (*i*.*e*., neurons, astrocytes and oligodendrocytes) but they are rarely co-expressed in the same cell [22]. Indeed, the distribution of creatine among brain regions relies on GAA synthesis by cells containing AGAT, followed by GAA transport to cell types expressing GAMT [15]. More information is needed to understand the region-specific importance of creatine and GAA, including their effects on memory performance, and whether dietary supplementation modulates tissue levels in brain. Indeed, GAA has been shown to be more effective at enhancing creatine levels in skeletal muscle [23] and could provide intracranial substrate for creatine.

The porcine model of brain function is more comparable to humans than rodent models [24,25] and the use of memory by pigs to solve tasks has been well established (*e*.*g*., spatial memory) [26,27]. Moreover, exploratory behavior is natural in this species [28], and can be exploited to demonstrate object recognition memory [29], which can be manipulated to test spatial, episodic-like memory [30]. Because young, fast-growing pigs must synthesize the vast majority of their accrued creatine [31] at the same time as the brain is rapidly developing, we predicted that pigs at this pre-pubertal stage of development would be more sensitive to creatine supplementation. The objective of the present study was to determine the effects of GAA and creatine supplementation on their concentrations in specific brain regions and on cognition in pre-pubertal swine by looking at object recognition and spatial memory, and the duration of memory retention. GAA and creatine concentrations were surveyed in hippocampus, prefrontal cortex (PFC), cerebellum and caudate nucleus following GAA or creatine supplementation. We hypothesized that supplementation with guanidino compounds will increase their concentrations in brain regions associated with memory and will improve spatial memory performance.

## Materials and methods

### Animals

Thirty male Yucatan miniature pigs (14–16 weeks old) were obtained from the Memorial University of Newfoundland breeding colony. Supplemented pigs, weight-matched to **Control** pigs (n = 14), were either supplemented with creatine monohydrate (**+Cr** group) (n = 8) (200 mg/(kg∙d)) or equimolar GAA (**+GAA** group) (n = 8) (157 mg/(kg∙d)). Supplementation amounts were approximately equal to 20 g creatine/d in humans, which is commonly used during a ‘loading phase’ to rapidly increase muscle creatine levels [1,32]. Pigs were group-housed and fed twice daily a standard pig grower diet (based on wheat, barley and canola; Eastern Farmers Co-op, St. John’s, N.L., Canada), providing 67% of energy as carbohydrate, 12% as fat and 21% as protein. Because the only source of creatine in this grower diet is from 1% meat meal, the control diet provided only ~6 mg/kg•d creatine, which represents ~5% of creatine accrual of growing pigs [23,31]. GAA was not detectable by our methods, but in muscle, GAA is ~0.1% of creatine concentrations, so was negligible compared to supplementation levels. Supplemental creatine and GAA doses were mixed with moistened standard grower feed, and hand fed to experimental groups during the twice daily feedings for 18–19 d. Animals were weighed every 5 d to adjust supplementation amounts. The Institutional Animal Care Committee at Memorial University of Newfoundland approved all animal-handling procedures (Protocol Number 11-55-RB) in strict accordance with Canadian Council on Animal Care guidelines. Isoflurane anesthesia was used at sacrifice and all efforts were made to minimize suffering.

### Memory testing

**Fig 1:**
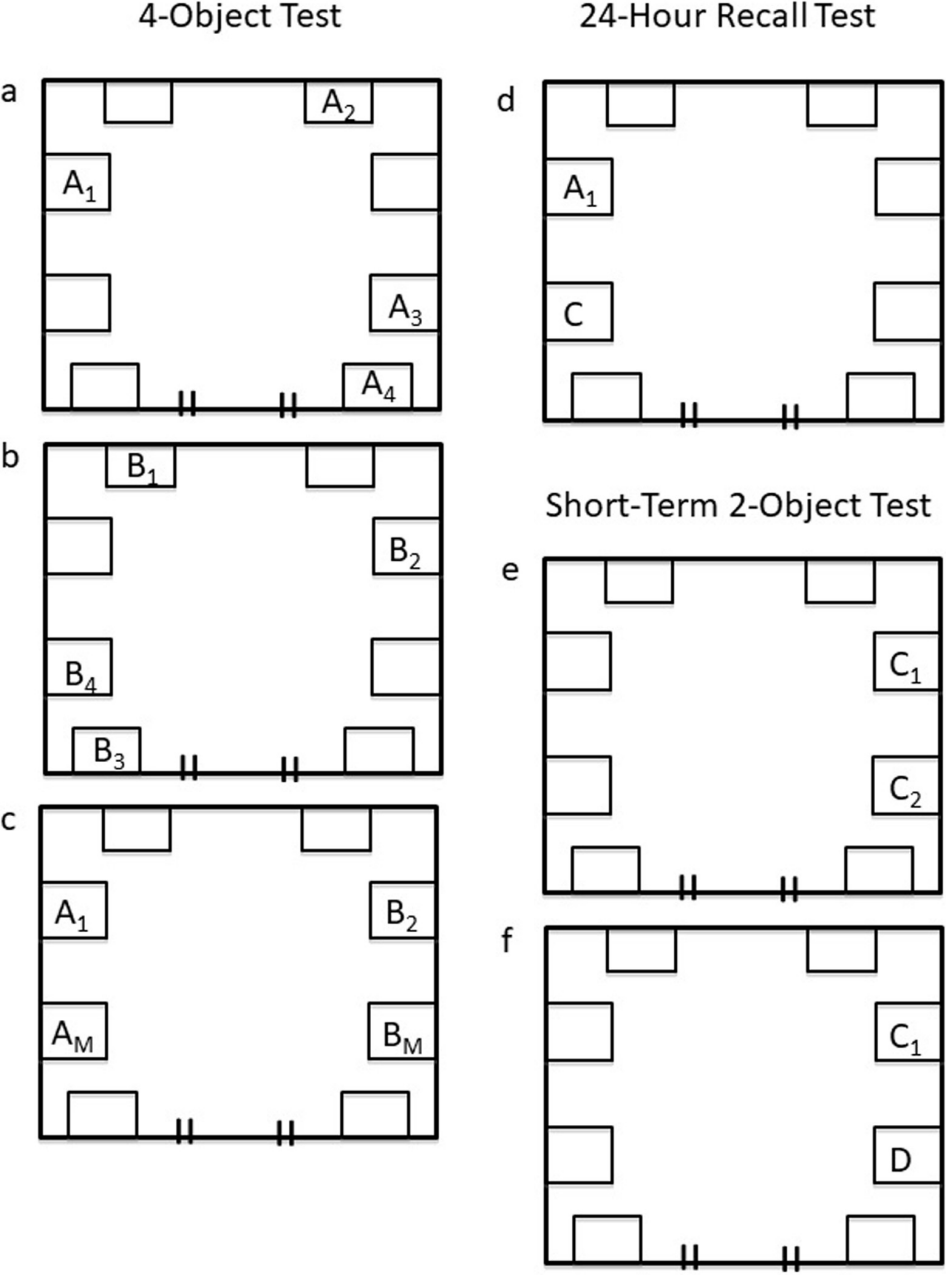
Schematic of testing apparatus. The testing apparatus was a 152 x 152 cm box with a latchable door. The rectangles within the box perimeter represent sites where objects could be attached for pig exploration. ***Fig 1a-c*: *4-Object Test***. The initial exposure is represented in (Fig 1a), the second exposure (Fig 1b) and the test exposure (Fig 1c). In (Fig 1c), subscript M denotes that this object has been moved from the previous exposure. ***Fig 1d*: *24-Hour Recall Test*.** During one episode, pigs were tested to distinguish between novel object C and object A (Fig 1d). Object A_1_ was viewed 24-h prior during the 4-object test in Fig 1c. ***Fig 1e-f*: *Short-Term 2-Object Test*.** The initial exposure (Fig 1e) and the test exposure (Fig 1f). In (Fig 1f), the object D is novel.

Initially, the test of object recognition/spatial memory in these pigs required habituation to a testing apparatus, which was a 152 cm x 152 cm box (90 cm height) with a latchable gate, and attachment locations for the fixture of various objects with a carabiner (Fig 1). Habituation was initiated by bringing the apparatus into the housing room each day during feeding for 10 d. Eventually, pigs were introduced into the box individually with the door unlatched, and then repeatedly with the door latched for 5 min. This occurred three times for each pig and confirmed object exploration in our apparatus. Food was placed in the box to promote a positive association with entering the testing apparatus, and various objects were latched in the box for pigs to explore. Researchers monitored pig behavior during box visits and discouraged jumping. The interior of the testing apparatus was washed with a high-pressure water hose to eliminate scent cues between animal visits. Memory testing was initiated on day 15 when researchers, who were blinded to experimental groups, recorded the time that pigs spent exploring 4 objects; all pigs were tested with all 4 objects at least once during the testing phase. For these tests, the objects are listed arbitrarily as: (A) a wooden coat hanger, (B) a can strainer, (C) a large metal spoon and (D) a hammer holder. All objects were selected at random and distributed such that object preference was controlled. Exploration was defined as the visual or physical orientation towards a spatially controlled object. The majority of exploration consisted of visual orientation with their snout within 10 cm of the object, but also consisted of rooting or manipulating the object with their snout and/or front hooves.

### 4-Object test

After the morning feed on day 15, the pigs entered the testing apparatus individually to complete a 4-object test (Fig 1A–1C). This test is based on one of the variations of the novel object recognition memory task described by [33]. Briefly, single pigs entered the box to initially explore for 5 min four object A’s that were fixed at specific locations within the apparatus (Fig 1A). The time that the pig explored each object was recorded, and after the pig exited the apparatus, the objects were removed and four object B’s were fixed at four novel locations (Fig 1B). The pig re-entered the box 50 min later and explored the object B's for 5 min. Before entering the apparatus for the final time 10 min later, researchers manipulated the objects to show pigs a combination of “older” object A’s and “recent” object B’s at either familiar (stationary) (*i*.*e*., A_1_ or B_1_) or novel (moved) locations (*i*.*e*., A_M_ or B_M_) (Fig 1C). Object exploration times were recorded for 5 min.

### 24-Hour recall test

After the morning feed on day 16, pigs were introduced one at a time into the testing apparatus to determine whether they recalled objects/locations from the previous day (Fig 1C). Pigs entered the apparatus containing two fixed objects; one object was seen the previous day and was in a familiar location (*e*.*g*., Object A_1_ from the 4-object test), and the other object was novel (*i*.*e*., Object C), which was also placed in a familiar (*i*.*e*., stationary) location (Fig 1D). Object exploration time was monitored for 5 min.

### Short-term 2-object test

After the morning feed on day 17, pigs explored the testing apparatus with two identical object C's at fixed locations for 5 min (Fig 1E). Pigs were tested 20 min later when researchers replaced one of the objects with novel object D (*i*.*e*., novel object in stationary location) and each pig was allowed to explore for 5 min (Fig 1F).

### Tissue collection

On day 18–19, pigs received their morning feed and were transported by truck to the small animal care facility on the Memorial University campus in covered crates. Anesthesia was induced with isoflurane (1–2%) in oxygen (1.5 L/min) within 1.5 h of feeding. Blood was sampled from the jugular vein and the liver was rapidly removed to euthanize the animal by exsanguination. The top of the skull was removed by making cuts along the saggital and coronal planes of the cranium with a saw. Brains were carefully removed and wet weights obtained. Using ice-cold instruments, the intact cerebellum was removed, followed by one entire hemisphere (side was counterbalanced across both the control and supplementary fed pig groups); both structures were placed in bags and frozen in liquid nitrogen, the remaining hemisphere was placed in a plastic tray on ice and the hippocampus, PFC and caudate nucleus were quickly excised and frozen in liquid nitrogen (excision techniques based on [34]). Tissues were stored at -80°C until analysis.

### Tissue and plasma analyses

Total creatine and GAA concentrations were measured in brain tissues (250 mg wet weight) and plasma (200 μL) using HPLC after derivatization with ninhydrin [35]. Homogenized tissues were incubated in buffer for 30 min to allow phosphocreatine to convert to creatine. Prior to each analysis, the entire sampled region was pulverized so that reported concentrations are a representative average of the entire brain region. Creatine kinase activity in cerebellum and PFC was measured using a commercial enzyme-linked immunoassay kit (BioVision Inc., Milpitas, CA, USA) with protein concentration measured by a bicinchoninic acid assay kit (Thermo Fisher Scientific, Waltham, MA, USA). Creatine kinase maximal reaction velocity was calculated as the product of specific activity and creatine concentration within each tissue. Because there were no differences among treatments for protein per g wet tissue in cerebellum (p = 0.42) or PFC (p = 0.47), metabolite and enzyme data were expressed per wet weight.

### Statistics

To estimate our sample size a priori, we used variance estimates for exploration time from a previous study in this herd of pigs using similar memory tests [30]. To detect a 33% change in exploration time, we calculated a sample size of 5 pigs per group; n = 8 was powered to detect a 20% difference. For metabolite analyses, using variance estimates from brain creatine and GAA analyses in young piglets from our laboratory, an outcome different of 33% needed 6–7 pigs per group. Metabolite concentrations, enzyme activity / maximal reaction velocity and pig weights were compared by one-way ANOVA and Tukey’s post-hoc test, where appropriate. A mixed ANOVA was used to compare the effects of GAA and creatine supplementation on the ability to recall recently experienced objects and their spatial configuration. Sphericity was not assumed and Greenhouse-Geisser adjusted degrees of freedom were selected for these comparisons. Regression analysis was performed to identify relationships between creatine or GAA concentrations (per gram of tissue) in brain regions and exploratory behavior. One-way ANOVA and regression analyses were performed with Prism software 5.0b. Mixed ANOVA was computed with SPSS software (Version 15+). Values are means ± standard deviation and p < 0.05 was considered significant in all cases.

## Results

### Weight gain

Guanidino compound supplementation did not cause discernible signs of stress and there was no overt effect of these compounds on weight gain relative to **Control** (Table 1). However, Tukey’s pairwise comparisons revealed that the **+GAA** pigs exhibited higher % weight gain compared to **+Cr** pigs from day 0–18 (p < 0.05).

**Table 1:** Pig weights on day 0, 5, 10, 15 and 18 and percent weight gain from day 0–18.^1^^,^^2^

	Day 0	Day 5	Day 10	Day 15	Day 18	Day 0–18
	kg					%
**Control**	12.8 ± 1.9	13.8 ± 1.7	14.5 ± 1.8	15.4 ± 2.0	16.4 ± 2.1	29.2 ±9.1^ab^
**+GAA**	12.3 ± 2.0	13.5 ± 1.9	14.1 ± 2.0	15.5 ± 2.4	16.4 ± 2.2	33.7 ± 7.1^a^
**+Cr**	13.2 ± 1.4	13.9 ± 1.3	14.9 ± 1.4	15.3 ± 1.5	16.2 ± 2.2	22.6 ± 3.4^b^

^1^Data expressed as mean ± SD. Pigs were supplemented for 18–19 days with 157 mg GAA/(kg∙d) (**+GAA**) (n = 8) or with 200 mg Cr/(kg∙d) **(+Cr**) (n = 8); **Control** pigs were unsupplemented (n = 14).

^2^Weight gain is expressed as percentage of initial weight on day 0 (mean ± SD); letters within a column indicate statistical difference (p < 0.05).

### GAA and creatine concentrations in plasma and brain

Plasma GAA and creatine concentrations were elevated by their respective supplementation, and **+GAA** pigs had greater plasma creatine *versus* **Control** pigs, but lower plasma creatine than **+Cr** pigs (p < 0.05) (as previously reported [23]) (Table 2). Method imprecision (CV) was 18% for creatine and 5% for GAA. Creatine supplementation did not affect GAA or creatine concentrations in any brain region; however, cerebellum was sensitive to GAA supplementation as the **+GAA** group had more GAA/g than **Control** and **+Cr** groups (p < 0.05) (Table 2). In the PFC of **+GAA** pigs, compared to **+Cr** pigs, creatine concentration was significantly greater (p < 0.05) and the GAA concentration was lower (p < 0.05).

**Table 2:** GAA and creatine concentrations in brain regions of pigs supplemented with GAA (+GAA) or creatine (+Cr) relative to unsupplemented Control.^1^^,^^2^

	Control	+Cr	+GAA	Control	+Cr	+GAA
	*μM GAA*			*μM creatine*		
Plasma	4.3 ± 1.4^a^	4.1 ± 1.1^a^	49.0 ± 45.4^b^	112 ± 45^a^	264 ± 59^b^	186 ± 37^c^
	*nmol GAA/g tissue*			*μmol creatine/g tissue*		
PFC	28.3 ± 13.4^ab^	38.0 ± 21.1^a^	13.4 ± 3.9^b^	5.6 ± 1.6^ab^	4.6 ± 1.3^a^	7.5 ± 3.5^b^
Cerebellum	24.2 ± 14.0^a^	21.6 ± 8.8^a^	58.6 ± 27.4^b^	9.8 ± 3.0	8.2 ± 2.9	9.5 ± 5.3
Hippocampus	29.5 ± 19.0	22.0 ± 6.8	31.43 ± 12.0	8.9 ± 4.0	7.0 ± 1.8	6.4 ± 1.9
Caudate Nucleus	28.0 ± 17.0	31.7 ± 20.7	22.9 ± 7.8	6.9 ± 2.0	7.9 ± 2.4	7.1 ± 4.6

^1^Data expressed as mean ± SD. Pigs were supplemented for 18–19 days with 157 mg GAA/(kg∙d) (**+GAA**) (n = 8) or with 200 mg Cr/(kg∙d) **(+Cr**) (n = 8); **Control** pigs were unsupplemented (n = 14).

^2^Means with different superscripts are significantly different within a row (p < 0.05).

### Creatine kinase activity

Creatine kinase activity was not affected by supplementation, although a trend (p = 0.10) was observed for cerebellum (Table 3). Method imprecision (CV) was 10% for creatine kinase activity. Creatine kinase maximal reaction velocity was significantly higher in PFC for **+GAA** pigs (p < 0.05).

**Table 3:** Creatine kinase (CK) activity and maximal reaction velocity in brain regions of pigs supplemented with GAA (+GAA) or creatine (+Cr) relative to unsupplemented Control.^1^^,^^2^

	Control	+Cr	+GAA
*CK Activity*	*mU/mg tissue*		
PFC	14.9 ± 12.5^ab^	13.6 ± 7.3^a^	25.4 ± 15.8^b^
Cerebellum^3^	2.62 ± 0.86	3.20 ± 0.75	2.21 ± 0.60
*CK Maximal Reaction Velocity*	mU/mg * μmol creatine/g tissue		
PFC	58.3 ± 36.7^a^	58.9 ± 29.0^a^	129.2 ± 44.3^b^
Cerebellum	20.3 ± 11.8	31.2 ± 7.74	23.5 ± 15.4

^1^Data expressed as mean ± SD. Pigs were supplemented for 18–19 days with 157 mg GAA/(kg∙d) (**+GAA**) (n = 6–7) or with 200 mg Cr/(kg∙d) **(+Cr**) (n = 5–7); **Control** pigs were unsupplemented (n = 5–7).

^2^Means with different superscripts are significantly different within a row (p < 0.05).

^3^p = 0.10

### Memory tests

#### 4-Object test

The total time pigs spent exploring objects was not affected by guanidino compound supplementation (F(2,26) = 0.036, p > 0.1; ε = -0.074). The average time devoted to object exploration during the 4-object test was 150.6 ± 70.2 seconds in the **+Cr** pigs, 150.0 ± 76.6 seconds in the **+GAA** pigs and 156.9 ± 55.7 seconds in the **Control** pigs. These exploration times represent ~50% of the total time spent in the testing apparatus.

A 3 x 2 x 2 ANOVA (diet group x object location stationary vs. moved x familiar (A) vs. novel (B) object) was carried out on absolute exploration times of each object type during the test phase. The purpose of this test was to determine whether supplemented groups were better able to discern between an older object (*i*.*e*., Object A) *versus* a more recently-viewed novel object (*i*.*e*., Object B), as well as if the pigs could recognize that objects were moved (*i*.*e*., A_M_ and B_M_). One pig was excluded from the **+GAA** group due to its escape from the apparatus. The three groups spent similar amounts of time with each of the four objects (F(2,25) = 0.065; p > 0.1) and there was no differential treatment of older *versus* more recent novel objects as a function of group (F(2, 25) = 0.023; p > 0.1), nor was there a group x object location (moved *versus* stationary) interaction (F (2,25) = 0.552; p > 0.1). However, at test, all pigs explored older objects (46.7 ± 29.2 seconds) more than recently-viewed objects (29.9 ± 23.5 seconds) (F(1,25) = 4.79, p < 0.01; ε = 0.161) (Table 4) and they spent more time exploring objects that were in moved locations (*i*.*e*., A_M_+B_M_) compared to objects that appeared in stationary locations (*i*.*e*., A_1_+B_1_) (Table 5) (F(1,25) = 7.80; p < 0.05, ε = 0.238).

**Table 4:** Summary of 3 x 2 x 2 ANOVA (diet group x object movement x older *versus* recent object) results of 4-object exploration using the Greenhouse-Gassier adjustment.^1^

	Degrees of freedom	F-value	Estimated epsilon (ε)
Older (A) versus recent (B) objects	1	4.79*	0.161
A versus B x Groups	2	0.08	0.006
Error (A versus B)	25	NA	NA
Stationary (A_1_, B_1_) versus moved (A_M_, B_M_)	1	7.80*	0.238
Stationary versus moved x Diet groups	2	0.552	0.042
Error (stationary versus moved)	25	NA	NA
A versus B x Stationary versus moved	1	0.662	0.026
A versus B x Stationary versus moved x Diet groups	2	1.30	0.094
Error (A versus B x Stationary versus moved)	25	NA	0.0

^1^Significance is indicated by * (p < 0.01).

**Table 5:** Total exploration times of previously viewed objects that were either stationary or moved during the 4-object test (F(1,25) = 7.805, p <0.01; ε = 0.238).^1^

	Exploration time (seconds)
Stationary Objects A_1_, B_1_	30.0 ± 5.5
Moved Objects A_M_, B_M_	46.8 ± 10.8

^1^ Pig groups were pooled (N = 29) and are presented as means ± SD.

#### 24-Hour recall test

A 3 x 2 mixed ANOVA (diet group x older *versus* recently-viewed object) compared performance on the 24-hour recall test. There was no effect of diet on total object exploration time in this test (F(2,26) = 0.235, p > 0.1; ε = 0.018); mean exploration times were 61.3 ± 44.9 seconds in **+Cr** pigs (n = 8), 33.1 ± 14.7 seconds in **+GAA** pigs (n = 8) and 46.3 ± 40.2 seconds in the **Control** pigs (n = 14). Overall, pigs did not show different exploration times for either object after 24 h, as the time spent with recently viewed and older objects was not different (F(2, 26) = 1.19, p > 0.1; ε = 0.084).

#### Short-term 2-object test

A 3 x 2 ANOVA was performed for the short-term 2-object test (diet group x older (C) *versus* recently-viewed (D) object) (Fig 1E and 1F). Pigs spent more time exploring the more novel object in this test (F(1,24) = 20.782, p < 0.01; ε = 0.464), which is in agreement with the findings of [30]. However, there was no effect of guanidino compound supplementation on identifying the more recent object during this short-term memory test (F(2,24) = 1.009, p > 0. 1; ε = 0.078). Once again, pigs in the three conditions spent a similar amount of time exploring objects (F(2,26) = 1.202, p > 0.1 ε = 0.078).

### Regression analysis

**Fig 2:**
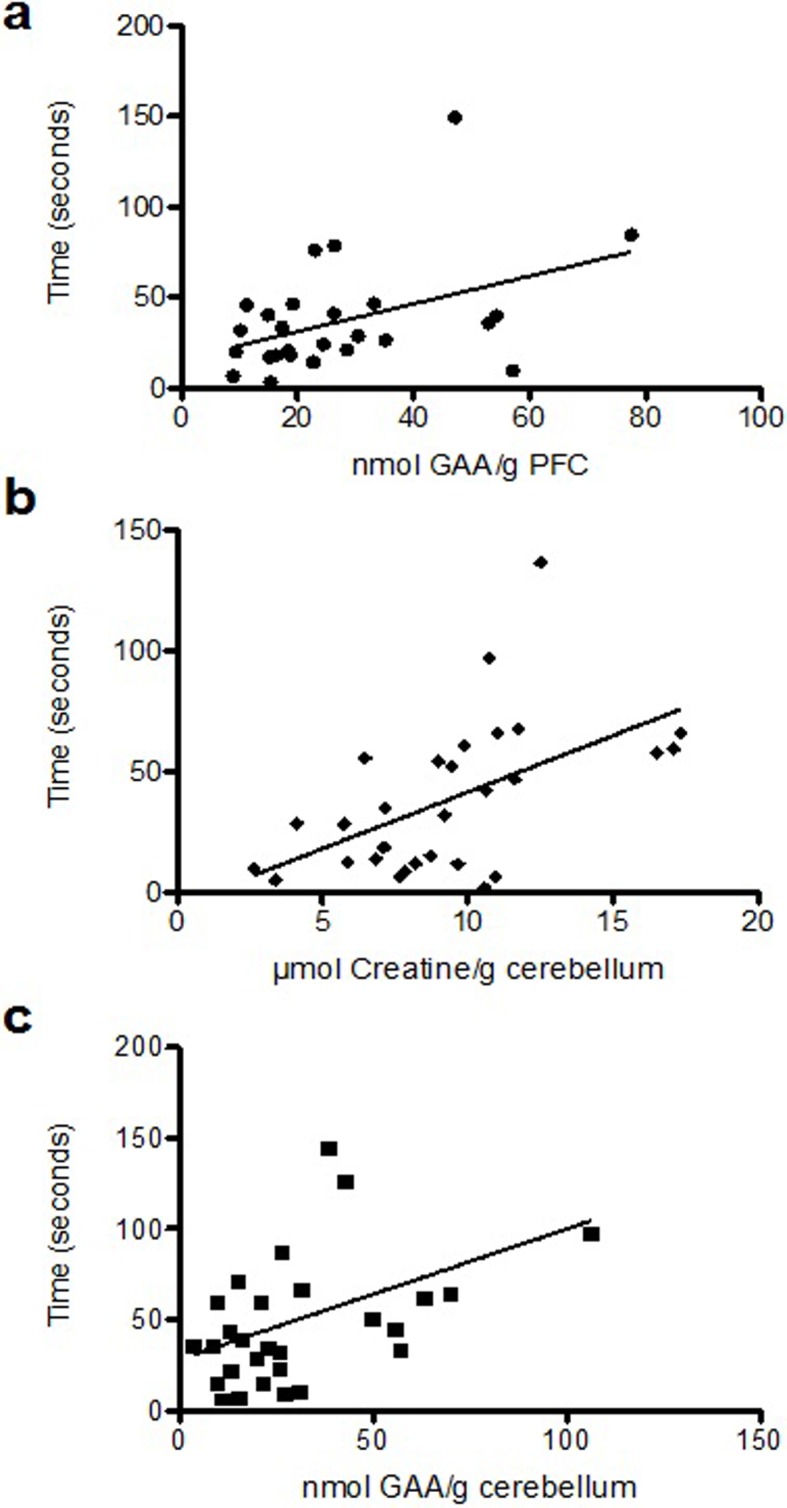
Regressions between brain guanidino compounds and performance. Regression analyses identified significant relationships between concentrations of GAA in the PFC and time exploring a familiar object during the 4-object test (Fig 2a), creatine in the cerebellum and time exploring a moved object during the 4-object test (Fig 2b) and GAA in the cerebellum and time exploring a novel object during the 24-hour recall test (Fig 2c).

Absolute exploration times of specific objects were correlated with concentrations of GAA and creatine in brain regions (S1 Table). Because there was no effect of supplementation on spatial and object recognition memory (p > 0.05), creatine and GAA concentrations of all pigs were pooled across study groups for each brain region. In the 4-object test, animals with a higher concentration of GAA in the PFC spent more time with the older stationary object (*i*.*e*., Object A_1_ in Fig 1C) (r = 0.59, F(1,25) = 5.4, p = 0.02) (Fig 2A), although this association appeared dependent on outlier data. The time that pigs explored the more recently observed moved object (*i*.*e*., Object B_M_ in Fig 1C) was positively correlated with creatine concentration in cerebellum (r = 0.61, F(1,25) = 11.2, p = 0.0002) (Fig 2B). Lastly, during the 24-h test, the GAA concentration of cerebellum was positively correlated with the time spent exploring a new object after 24 h (*i*.*e*., Object C in Fig 1D) (r = 0.53, F(1,25) = 5.6, p = 0.02) (Fig 2C).

## Discussion

The study objective was to determine whether supplemental GAA or creatine reaches the brain, and if either guanidino compound was associated with improved memory in pigs. Despite no effect of supplementation on pig memory tests, GAA and creatine concentrations in different regions of pig brain correlated with memory performance. Indeed, this study demonstrates that cerebellum and PFC are important sites of GAA and creatine metabolism and regulation in the pig brain with relevance to spatial and object recognition memory.

The present study confirmed the findings of Kouwenberg et al. [30] for pig performance in a short-term 2-object memory test. It was further shown that memory persists longer than 20 minutes in pigs, and that they can recall when and where they experienced up to four objects after 65 min. However, pigs do not seem to exhibit this memory after 24 h, using the present testing methods, and the two objects were explored much less (total exploration time) compared to four objects. The robustness and limitations of these methods to assess pig memory may prove useful to others aiming to identify biological or pharmacological agents with psychoactive properties in a relevant animal model.

Memory performance was associated with creatine and GAA concentrations in cerebellum. The creatine concentration of the cerebellum and the ability to recognize the moved object during the 4-object test revealed a significant correlation. Moreover, the cerebellum had more creatine per gram than other sampled brain regions, and pigs successfully identified moved objects during that test. Furthermore, the GAA concentration of cerebellum was positively associated with time spent exploring the novel object in the 24-hour recall test, which is interesting because pigs did not preferentially explore the novel object in that test, and the cerebellum was the only region sensitive to GAA supplementation. Moreover, GAA supplementation led to higher GAA concentration, but not creatine concentration or creatine kinase activity or maximal reaction velocity in the cerebellum. Together, these data suggest that the cerebellum is a region of GAA uptake, but not creatine synthesis, which is in contrast to imaging data in GAA-supplemented men who appeared to accumulate creatine in the cerebellum [36]. The accumulation of GAA in cerebellum did not negatively impact creatine kinase activity, although more research is needed to know whether GAA uptake is a positive outcome in the absence of creatine synthesis. Regardless, the proximity of cerebellum to the carotid artery makes it a logical site of creatine and GAA uptake from the circulation, and it is expected that the ability of GAA to traverse the blood-brain barrier and reach the cerebellum in **+GAA** pigs was likely mediated by the higher plasma GAA concentrations with supplementation and the sensitivity of CT1 or GAA2 [37]. The cerebellum is activated during memory recollection in humans [38] and new ideas regarding its role in cognition are being considered [39], so the association between creatine metabolism in the cerebellum and a short-term memory task in pigs is intriguing. Creatine metabolism may be critical in the cerebellum in order to support proper brain function and memory consolidation.

There was also a positive association between GAA concentration in the PFC and the exploration of a previously seen object during the 4-object test (*i*.*e*., A_1_). It is difficult to discern whether GAA in the PFC was beneficial or detrimental in this case because GAA concentration was *lower* in the PFC of GAA-supplemented animals compared to **+Cr** pigs. Indeed, there are studies that describe negative effects of GAA administration [40]. While long-term GAA supplementation was safe in men, the ability of GAA to bind to GABA receptors and low basal levels may mean that supplemental GAA can have variable effects on brain. Associations between memory and the PFC are plausible because the PFC contributes to working memory [41] and fear extinction [42]. Interestingly, the lower GAA concentration with GAA supplementation was concomitant with a greater creatine concentration in the PFC, compared to creatine-supplemented pigs. Moreover, GAA supplementation led to a higher creatine kinase activity and maximal reaction velocity in PFC, perhaps explaining the association of PFC GAA concentration on memory performance in this test. Alternatively, these associations could be related to some other function of GAA and creatine, including as compatible osmolyte, a cellular antioxidant or as a neurotransmitter influencing GABAergic and glutamatergic neurotransmission [43]. These data implicate the PFC as a regulatory site of GAA metabolism in the pig brain, as GAA may be distributed by this tissue after peripheral uptake by cerebellum. It has been shown that neurons from the dentate nucleus of the cerebellum project to the PFC (*via* the thalamus) in non-human primates [44], and imaging studies show co-activation of these regions during cognitive tasks [45]. Further investigations into a potential cerebellum-PFC axis for the metabolism of creatine and other intermediates are warranted.

Creatine concentration varied among brain regions of these 16-18-week-old pigs. Interestingly, creatine concentration in the cerebellum and hippocampus of these pigs was 35–40% greater than in the entire brains of 7-11-d-old domestic pigs [31]. Indeed, this accumulation in these regions could reflect higher transport of creatine as CT1 is abundant in Purkinje cells of the cerebellum and dentate gyrus of the hippocampus of rat brain [46]. The cerebellum [38] and hippocampus [47] are critical for advanced cognitive processes, and we postulate that pigs have an increased reliance on phosphocreatine-derived ATP for memory consolidation in these regions upon full development. The cerebellum is not fully developed until ~16 y in men (~12 y in women), which coincides with our current late pre-pubertal pig model (sexual maturity occurs at 4–6 mo of age in Yucatan miniature swine [48]). It is possible that some of our pigs were less mature than others with respect to cerebellum development, which could contribute to variability in our results. The interaction of this region with episodic-like memory and its sensitivity to GAA supplementation suggests an importance of creatine accrual in this region. Hippocampal development fits a cubic growth trajectory in humans that maximizes at 9–11 years of age [49], and the growth pattern of this tissue is matched by declining hippocampal neurogenesis [50]. Neurogenesis is supported by oxidative metabolism, whereas glycolysis is the major ATP source in brain [51], and the importance of phosphocreatine for brain function may depend on the developmental stage [52]. The importance of phosphocreatine to support anaerobic brain function is available in studies describing GAA and creatine as neuroprotective substrates during ischemic episodes [10,11]. Furthermore, creatine may support cognitive function during stress in human [53] and animal studies [20,21]. However, it is not clear whether this increased support was due to enhanced anaerobic demands in brain, as chronically supplemented mice had enhanced in vitro oxidative phosphorylation in hippocampal mitochondria [9]. Further studies might investigate the ontogeny of brain creatine metabolism *in vivo* in the more translational pig model.

Although we observed interesting associations between creatine and GAA in PFC and cerebellum with memory performance, we did not observe significant effects of supplementation on any tests and creatine supplementation did not change creatine in any brain region. Our doses were based on 100% creatine accrual rates for pigs [31], so were sufficient even without endogenous synthesis of creatine. In an unpublished study in 2-week-old piglets using this dose, we found higher creatine concentrations in most tissues with 7 days of supplementation, except in muscle and brain. Although we extended the supplementation period in the current study to 18–19 days and in spite of much higher plasma and tissue creatine concentrations [23], no brain region had higher creatine with creatine supplementation. It is possible that an even longer time period is required to enhance brain creatine levels. On the other hand, it is also possible that creatine supplementation might only work on brain performance when deficient in the first place, or when challenged with various stressors [8, 10,11].

An unexpected finding was that **+GAA** pigs exhibited greater % weight gain over the course of the study than creatine-supplemented pigs. GAA has been shown to spare arginine and increase weight gain in chicks fed arginine-deficient diets [54], and so it is possible that GAA partially spares arginine in young pigs to increase growth. However, our grower feed was not predicted to be low in arginine, and a more controlled study focused on growth and arginine requirement is needed to confirm this observation.

This study reports that supplemental creatine and GAA were unable to improve memory performance in young pigs. It is possible that the effects of supplemental creatine or GAA on memory are only observed with a habitual creatine-deficient diet, as has been shown in human vegetarians [3]. However, this study did demonstrate that the cerebellum and the PFC were important sites of creatine metabolism in the pig and that GAA levels in the PFC and creatine levels in the cerebellum were positively associated with memory performance. The description of creatine and GAA among brain regions provides new insight into the importance of creatine metabolism during brain development. How creatine and GAA are metabolized in these various regions requires elucidation in order to understand their role in cognitive performance and recovery.

## Supporting information

S1 Table Regression parameters for associations between tissue concentrations of GAA and creatine in pig brain regions and performance on the 4-object test. (DOCX)

## Abbreviations

AGAT: arginine:glycine amidinotransferase
CT1: creatine transporter 1
GAA: guanidinoacetic acid
GAA2: γ-aminobutyric acid transporter 2
GAMT: guanidinoacetate methyltransferase
PFC: prefrontal cortex
