# Supplementary material for: Effects of supplemental creatine and guanidinoacetic acid on spatial memory and the brain of weaned Yucatan miniature pigs
Source: PLoS One. 2020 Jan 6;15(1):e0226806. doi: 10.1371/journal.pone.0226806 (PMC6944358; doi:10.1371/journal.pone.0226806)
Supplement: S1 Table — (DOCX) [file pone.0226806.s001.docx]

**Supporting information**

**S1 Table.** **Regression parameters for associations between tissue concentrations of GAA and creatine in pig brain regions and performance on the 4-object test.^1^**

| Tissue parameter | Regression Parameter | Exploration of object A1 | Exploration of moved object Am | Exploration of object B1 | Exploration of moved object BM | Total exploration time |
| --- | --- | --- | --- | --- | --- | --- |
| [GAA] in PFC | r | 0.42 | 0.26 | 0.22 | 0.05 | 0.25 |
|  | P-value | 0.03 | 0.20 | 0.26 | 0.79 | 0.20 |
|  | Slope | 0.77 | 0.62 | -0.31 | -0.10 | 0.98 |
| [Creatine] in PFC | r | 0.03 | 0.18 | 0.14 | 0.10 | 0.20 |
|  | P-value | 0.87 | 0.36 | 0.49 | 0.61 | 0.32 |
|  | Slope | -0.40 | 3.04 | 1.31 | 1.31 | 5.26 |
| [GAA] in cerebellum | r | 0.09 | 0.10 | 0.13 | 0.20 | 0.17 |
|  | P-value | 0.65 | 0.60 | 0.51 | 0.31 | 0.38 |
|  | Slope | -0.12 | 0.19 | 0.13 | 0.27 | 0.48 |
| [Creatine] in cerebellum | r | 0.28 | 0.00 | 0.18 | 0.54 | 0.20 |
|  | P-value | 0.14 | 0.99 | 0.35 | 0.00 | 0.30 |
|  | Slope | -2.31 | -0.03 | 1.14 | 4.67 | 3.47 |
| [GAA] in hippocampus | r | 0.08 | 0.01 | 0.18 | 0.07 | 0.06 |
|  | P-value | 0.70 | 0.97 | 0.36 | 0.73 | 0.75 |
|  | Slope | 0.15 | -0.02 | 0.28 | -0.14 | 0.26 |
| [Creatine] in hippocampus | r | 0.01 | 0.19 | 0.16 | 0.07 | 0.04 |
|  | P-value | 0.96 | 0.32 | 0.39 | 0.73 | 0.82 |
|  | Slope | 0.09 | -2.60 | -1.20 | -0.65 | 0.89 |
| [GAA] in caudate nucleus | r | 0.05 | 0.09 | 0.30 | 0.13 | 0.25 |
|  | P-value | 0.82 | 0.65 | 0.12 | 0.50 | 0.21 |
|  | Slope | -0.09 | -0.24 | -0.44 | -0.21 | -0.97 |
| [Creatine] in caudate nucleus | r | 0.03 | 0.27 | 0.00 | 0.05 | 0.21 |
|  | P-value | 0.89 | 0.17 | 0.99 | 0.80 | 0.27 |
|  | Slope | 0.27 | 3.88 | 0.02 | 0.43 | 4.59 |

^1^ Outcomes for the test were the time animals spent exploring objects in a familiar location (*i.e.*, A1 and B1) and moved locations (*i.e.,* Am and Bm) as well as exploratory behavior (*i.e.,* total exploration time).
